# Identification of *Eimeria tenella* sporozoite immunodominant mimotopes by random phage-display peptide libraries–a proof of concept study

**DOI:** 10.3389/fvets.2023.1223436

**Published:** 2023-07-24

**Authors:** Marco A. Juárez-Estrada, Guillermo Tellez-Isaias, Danielle M. Graham, Lauren Laverty, Amanda Gayosso-Vázquez, Rogelio A. Alonso-Morales

**Affiliations:** ^1^Departamento de Medicina y Zootecnia de Aves, Facultad de Medicina Veterinaria y Zootecnia, Universidad Nacional Autónoma de México, Mexico City, Mexico; ^2^Departamento de Genética y Bioestadística, Facultad de Medicina Veterinaria y Zootecnia, Universidad Nacional Autónoma de México, Mexico City, Mexico; ^3^Department of Poultry Science, University of Arkansas, Fayetteville, AR, United States

**Keywords:** Apicomplexa, 2nd-generation merozoites, reverse immunology, Et-MIC4, TRAP-family, Et EF-2, beta-dynein chain, ankyrin-repeat

## Abstract

**Introduction:**

Coccidiosis, caused by parasites of numerous *Eimeria* species, has long been recognized as an economically significant disease in the chicken industry worldwide. The rise of anti-coccidian resistance has driven a search for other parasite management techniques. Recombinant antigen vaccination presents a highly feasible alternative. Properly identifying antigens that might trigger a potent immune response is one of the major obstacles to creating a viable genetically modified vaccine.

**Methods:**

This study evaluated a reverse immunology approach for the identification of B-cell epitopes. Antisera from rabbits and hens inoculated with whole-sporozoites of *E. tenella* were used to identify Western blot antigens. The rabbit IgG fraction from the anti-sporozoite serum exhibited the highest reactogenicity; consequently, it was purified and utilized to screen two random Phage-display peptide libraries (12 mer and c7c mer). After three panning rounds, 20 clones from each library were randomly selected, their nucleotide sequences acquired, and their reactivity to anti-sporozoite *E. tenella* serum assessed. The selected peptide clones inferred amino acid sequences matched numerous *E. tenella* proteins.

**Results and Conclusions:**

The extracellular domain of the epidermal growth factor-like (EGF-like) repeats, and the thrombospondin type-I (TSP-1) repeats of *E. tenella* micronemal protein 4 (EtMIC4) matched with the c7c mer selected clones CNTGSPYEC (2/20) and CMSTGLSSC (1/20) respectively. The clone CSISSLTHC that matched with a conserved hypothetical protein of *E. tenella* was widely selected (3/20). Selected clones from the 12-mer phage display library AGHTTQFNSKTT (7/20), GPNSAFWAGSER (2/20) and HFAYWWNGVRGP (8/20) showed similarities with a cullin homolog, elongation factor-2 and beta-dynein chain a putative *E. tenella* protein, respectively. Four immunodominant clones were previously selected and used to immunize rabbits. By ELISA and Western blot, all rabbit anti-clone serums detected *E. tenella* native antigens.

**Discussion:**

Thus, selected phagotopes contained recombinant *E. tenella* antigen peptides. Using antibodies against *E. tenella* sporozoites, this study demonstrated the feasibility of screening Phage-display random peptide libraries for true immunotopes. In addition, this study looked at an approach for finding novel candidates that could be used as an *E. tenella* recombinant epitope-based vaccine.

## Introduction

Avian coccidia belongs to the Eimeriidae family and the phylum Apicomplexa (1). *Eimeria tenella* is one of the most pathogenic species of avian coccidiosis, causing massive economic damage to the global poultry industry (2, 3). Several live vaccines consisting of either virulent or attenuated coccidian strains have been commercially developed in recent years (4). Live oocyst vaccines are a limited but useful option to prophylactic medicine; however, a recombinant vaccine with specific parasite antigens that develop strong protective coccidia-immunity would be preferable (5, 6). Several studies have identified potential protective antigens from *Eimeria* such as AMA1, EF-1α, EF-2, MIC-1, MIC-2, MIC-3, IMP-1, LDH1, SAG1, Gam22, Gam 56, Gam 82, Rhomboid-like Protein, Profilin and SO7, however, attempts to produce a successful commercial recombinant vaccine have been hindered until now (5–8).

Immune responses to *Eimeria* infections involve numerous aspects of innate and adaptive/acquired immunity (4). Although protective immunity to *Eimeria* includes both cellular and humoral immune pathways, it is commonly assumed that the primary role is based on a robust cell-mediated response, with antibodies presumably playing a minor role (4, 9). Nonetheless, it appears that antibodies play a significant role in protection under specific conditions (10–12). Class B epitopes have been found in all seven species of chicken coccidia, indicating that this antigen class may protect chickens from coccidiosis (8, 11, 13). As a result, an effective recombinant vaccine against coccidiosis should contain both lymphocyte type T and B antigens to elicit a successful cellular immune response (6–8). However, a better definition of protective B-cell epitopes from *Eimeri*a species is still needed (14). It is critical to discover the involvement of specific proteins from different life-cycle stages of the *Eimeria*, possibly those engaged in the earliest steps of invasion and all those associated with pathogenesis and parasite survival (15, 16).

The main objective of epitope identification is to replace complex antigens used for immunization programs, antibody production, and even serodiagnosis (17–19). Additionally, with an appropriate methodology, it is easier to find a peptide that can induce either antibody production or T-cell induction (8, 11, 14, 20, 21).

Phage-display technology is a low-cost, high-performance screening approach for identifying peptides with high molecular affinity to specific antibodies (17, 22). Epitope discovery is critical in diagnostics, immunotherapy, drug discovery, and vaccine development (17, 22, 23). This methodology enables the selection of mimotopes, peptides that replicate a pathogen’s native epitopes, even without prior knowledge of the natural ligand region (19). M13 Phage display vectors combine peptide gene sequences to coat protein genes, usually gIII or gVIII. Therefore, nucleotide sequencing of the phage DNA can be used to determine the amino acid sequence of the selected peptide (17). Rounds of screening (panning) can be used to select specific peptides with affinity to a ligand from Phage display libraries, exposing billions of peptides variants present in the libraries to an immobilized ligand and washing away unspecific phages, amplifying the bound phage by infecting *Escherichia coli* (17, 19).

The selection of peptides from random Phage display libraries by specific antibodies is an attractive strategy for the possible generation of pure epitope vaccines based on phagotopes (19, 21, 22). In recent years, screening Phage-display libraries with specific antibodies has become an attractive strategy for designing anticoccidial therapies to control this disease (9, 14, 24, 25).

Although monoclonal antibodies are excellent mimotope selectors (9, 14, 17, 26), polyclonal antibodies are generally favored because they are widely available and may find novel immunogenic epitopes (19, 22). Due to the pathogen’s thousands of years of co-evolution to avoid the immune response, epitope selection with the host’s polyclonal antiserum might be difficult (27, 28). An alternative approach to effective epitope identification is using antibodies generated in unnatural host species, where the hidden vital antigens for the natural host may be recognized, producing antibodies against them (18, 19, 21).

The phage display library technique is relatively new for peptide-based parasite vaccine development (18, 29), and it has never been used before in screening *E. tenella* sporozoite mimotopes for possible vaccinations against the disease. Therefore, in the present investigation, rabbit sera antibodies generated against sporozoites of *E. tenella* were used to screen two Phage display random peptides libraries in order to identify highly immunogenic epitopes involved in *E. tenella* infection. Using these heterologous antisera, we were able to identify highly immunoreactive sporozoite epitopes from *E. tenella*.

## Materials and methods

### Parasites

The wild-type strain of *E. tenella* used in this research was isolated from birds showing clinical signs of cecal coccidia on a broiler farm in Querétaro, Mexico’s central state. The oocysts of *E. tenella* were purified using the method reported by Stephan et al. (30). Three-week-old Leghorn Specific Pathogen Free (SPF) chickens (SPAFAS Inc., Norwich, CT, United States) were used to propagate oocysts. Following established procedures, cecal oocysts were isolated, sporulated, and cleaned (31).

### Sporozoites and second generation of merozoites antigens preparation

Sporocysts were released by vortexed sporulated oocysts (2.5 × 10^7^/ml) at 2,000 X g using 1 mm diameter glass beads (Sigma-Aldrich, Inc., Burlington, MA, United States) for 1 min. A 50% Percoll gradient (density 1.13 g/mL, GE Healthcare, Piscataway, NJ, United States) was used to purify sporocysts (32). 1 × 10^7^ purified sporocysts were resuspended in an excystation medium and incubated at 42°C for 150 min. PBS (pH 7.4) with 0.75% (w/v) taurodeoxycholic acid (Sigma-Aldrich, Inc., Burlington, MA, United States) and 0.25% (w/v) trypsin from porcine pancreas Type II-S (Sigma-Aldrich, Inc., Burlington, MA, United States) comprised the excystation medium. Sporozoites (Sz) were washed and purified using a 60% Percoll gradient (density 1.13 g/mL, GE Healthcare, Piscataway, NJ, United States). Sporozoites were resuspended in sterile PBS for the immunization program, gradually frozen at −70°C at a 1°C/min rate, and then stored at −70°C until use.

Three 10-week-old hybrid pullets were gavage-inoculated with 5 × 10^5^ sporulated oocysts of *E. tenella* and euthanized 112 h post-infection (PI) to collect second-generation merozoites (Mz). These birds’ intestines were treated for merozoite isolation, as described previously by Liu et al. (33). The merozoites were purified using the method outlined by Geysen et al. (34). Each parasite (Sz and Mz) was suspended in sterile PBS with a protease inhibitor (cOmpleteTM Roche Applied Science, Mannheim, Germany). Five freeze/thaw cycles each disrupted both asexual zoite stages. The final suspension was centrifuged at 2,000 x g for 18 min at 4°C. The supernatants were collected, and the protein concentration was measured with the Bradford reagent (BioRad, Hercules, CA, United States) using a bovine serum albumin standard curve (Sigma-Aldrich, Inc., Burlington, MA, United States). Antigen suspensions were kept in 200 μL aliquots at −70°C until needed.

### Rabbit and chicken antisera against whole sporozoites of *Eimeria tenella*

Two white New Zealand rabbits (2.5 kg) and two SPF White Leghorn chickens (1.1 kg) were immunized subcutaneously with 100 μg of purified whole sporozoites (5.3 × 10^6^ sporozoites), diluted 1:1 with the IMS 1313 N VG PR nanoparticle adjuvant (Seppic Montanide™, France) in a total volume of 1 mL as previously was described by Juárez-Estrada et al. (35). Three further immunizations were given at two-week intervals. Both rabbits were euthanized by exsanguination 1 week after the last immunization. After clotting for 1 h at room temperature and overnight at 4°C, the blood was centrifuged at 2000 X g for 5 min, and serum samples were aliquoted and stored at −20°C until use. Both SPF Leghorn chickens were wing-bled, and serum was extracted by clotting it for 1 h at room temperature and overnight at 4°C, then centrifuged at 2000 X g for 5 min, aliquoted, and stored at −20°C until needed. According to the manufacturer’s instructions, the rabbit serum’s IgG fraction was purified by affinity chromatography using Sepharose 4 FF protein G (GE HLS Marlborough, Mass., United States).

### Enzyme-linked immunosorbent assay (ELISA)

The reactogenicity of the bird’s and rabbit’s anti-sporozoite sera, the rabbit anticlone serum, and the purified rabbit IgG fraction to sporozoite and 2nd generation merozoite antigens were evaluated by ELISA, essentially, as Constantinoiu et al. (3) described it. 96-well microtiter plates (MaxiSorb, Nunc, Roskilde, Denmark) were coated overnight at 4°C with 1 μg of sporozoite or merozoite antigen in 100 μL of carbonate buffer (0.1 M sodium bicarbonate and 0.1 M sodium carbonate buffer, pH 9.6). The control wells were incubated with only 100 μL of carbonate buffer. After four washes on a shaker with a saline solution (S) (120 mM NaCl, 25 mM Tris–HCl, pH 7.9) containing 1% Tween 20 (ST), non-specific binding sites were blocked by incubating for 1 h at 37°C in a static oven with 110 μL of 5% skim milk in ST (STM). Sera diluted in STM (1:10 and 1:100) were added to the test and control wells after four ST washes, and both were then incubated for 1 h at 37°C. Each plate included negative control sera from unimmunized SPF Leghorn chickens. Following incubation, the plates were washed four times with ST and incubated with their respective secondary antibody peroxidase conjugate diluted 1:2000 with STM (anti-rabbit, anti-chicken) (Jackson ImmunoResearch Laboratories, Inc. West Grove, PA, United States). After 1 h at 37°C, the plates were washed four times. The enzymatic reaction was developed by adding 100 μL of OPD chromogen (o-phenylendiamine dihydrochloride SIGMA, St. Louis MO, USA) at 5 μg/10 mL in citrate buffer (0.1 M citric acid, 0.1 M sodium citrate p.H. 4.5, and 20 μL of 30% hydrogen peroxide) for 10 min in a shaker under dark conditions. An ELISA microplate spectrophotometer (Epoch, BioTek, Winooski, VT, United States) was used to read the absorbance produced by substrate hydrolysis at 450 nm. All serum samples were examined in duplicate thrice times.

### SDS-PAGE and Western blot

For SDS-PAGE analysis of both asexual zoite phases, 20 μg of each purified fraction was separated by 12% SDS-PAGE under standard reducing conditions. According to Constantinoiu et al. (36), the resolved proteins were stained with Coomassie brilliant blue (CBB) or electrotransferred on polyvinylidene difluoride membranes (PVDF) (Bio-Rad, Hercules, CA, USA). PVDF membranes were probed with anti-sporozoite of *E. tenella* sera from rabbits and Leghorn fowl. As a secondary antibody, horseradish peroxidase (HRP)-conjugated IgG goat anti-rabbit IgG, and (HRP)-conjugated IgG goat anti-chicken IgY (Jackson ImmunoResearch Laboratories, Philadelphia, PA, United States) were employed at a 1: 1500 (v/v) in 5% (w/v) STM. Tablets of DAB SigmaFast™ (3,3′-diaminobenzidine) (Sigma-Aldrich, St Louis MO, United States) at 10 mg/5 mL were used to visualize the PVDF membranes.

### Screening of a phage-display library and characterization of selected clones

IgG fraction purified from rabbits previously immunized with sporozoites of *E. tenella* was used to screen commercially available dodecapeptide (Ph.D.-12TM) and cysteine-constrained heptapeptide (Ph.D.-C7CTM) M13 pIII libraries (New England Biolabs Inc., Ipswich, MA, United States). Three rounds of clone selection were performed for each screening using progressively decreasing quantities of antibodies (20 μg, 10 μg, and 5 μg, respectively). Briefly, two wells in a 96-well polystyrene microtiter plate (Maxisorb, Nunc, Roskilde, Denmark) were coated and incubated at 4°C overnight (O/N) with IgG in sterile phosphate chloride buffer (PCB) (0.1 M potassium chloride, 3 mM sodium chloride, 5 mM sodium phosphate dibasic, 1 mM potassium phosphate monobasic). Wells were washed five times with 0.1% (v/v) Tween 20 in 0.01 M phosphate-buffered saline (TPBS), pH 7.2, then blocked with BSA (5 μg/mL in distilled H2O). Every plate was incubated for 1 h at room temperature with 1 × 10^10^ plaque-forming units (pfu) of each phage library. After washing with TPBS, the bound phages were eluted with 100 μL of 0.1 M glycine-HCl, pH 2.2 (hereafter referred to as “eluate”) and immediately neutralized with 15 μL of 1 M Tris–HCl, pH 9.1. The eluate phages were amplified and titer through infection of the *E. coli* strain ER2738 (New England Biolabs Inc., Ipswich, MA, United States). The phages were titled as plaque-forming units by ml (pfu/ml). For the next round of phage-display screening, every phage-display library eluate was amplified and concentrated with polyethylene glycol-8000 PEG/2.5 M NaCl (20%/40%) following the manufacturer’s instructions. The phage input and output titers ratio was used to calculate the specific phage enrichment level.

### Phage clone selection and nucleotide sequence analysis

An aliquot of approximately 100 pfu from the third-round panning was combined with *E. coli* ER2738 and plated on LB agar with IPTG (isopropyl-β-D thiogalactopyranoside) and X-gal (5-bromo-4-chloro-3-indoyl-β-D-galactopyranoside). After 37°C O/N growth, twenty separate isolated phage plaques per library were randomly collected (*n* = 40). Each plaque was grown in 2XYT broth containing a dilution of 1/1000 of *E. coli* strain ER2738 grown O/N and incubated for 5 h at 37°C at 250 rpm. Following standard procedures, single-stranded phage DNA was isolated using an iodide buffer extraction, precipitated with 1 volume of ethanol, and resuspended in nuclease-free distilled water (Thermo Fisher Scientific Inc. Waltham, MA, United States). Purified phage DNA was sequenced with the Big Dye Terminator v1.1 cycle sequencing kit (Thermo Fisher Scientific, Waltham, MA, United States) using the pIII primer (5’-CCAGACGTTAGTAAATG-3′).

### Bioinformatic analysis

Using the Clone Manager Professional Edition program, version 9.0 (Scientific and educational software, Cary, NC, United States), the amino acid sequences of the peptide were determined from the nucleotide sequences. The protein BLAST software^1^ was used to screen the selected clones in the published sequences of the *Eimeria tenella* genome. The settings for the search were: Database: non-redundant protein sequence (nr); organism: *Eimeria tenella* (taxid: 5802); algorithm: blast (protein–protein BLAST).

### Immune reactivity assessment of phage–displayed clones to rabbit and chicken anti-sporozoite serum by ELISA

ELISA reactogenicity to the selected phage clones was assessed using the pooled anti-sporozoite serum from both rabbits immunized with sporozoites of *E. tenella*. Briefly, phage–displayed clones were bound to microtiter plates (MaxiSorb, Nunc, Roskilde, Denmark) and developed with the anti-sporozoite sera diluted (1:10 and 1:100). Wild-type M13 phage and a clone of M13 phage (Ph.D. 12mer and c7c libraries) from screening with pseudorabies virus rabbit IgGs were used as negative controls. The total phage elution of the third round panning of both libraries was amplified and used as a positive control. After four washes with SS, the anti-sporozoite antibodies were detected using a secondary anti-rabbit IgG or anti-chicken IgY peroxidase conjugate diluted at 1:2,000 in blocking solution for 1 h at 37°C (Jackson ImmunoResearch Laboratories, Inc. West Grove, PA, United States). The plate was then washed four more times with SS before adding 100 μL of TMB chromogen at 1 mg/10 mL in citrate buffer (3,3′,5,5′-Tetramethylbenzidine, Sigma-Aldrich, Inc., Burlington, MA, United States), the addition of 2 N sulfuric acid stopped this reaction. An ELISA reader (Epoch, BioTek, Winooski, VT, United States) was used to determine the color intensity at 450 nm. Otherwise, the antisera reactivity of phage-immunized rabbits was determined in phage-ELISA format using 1 μg/well of *E. tenella* sporozoite and merozoite as antigens.

### Rabbit antisera against each selected phage clone

Rabbit antisera for every one of the four most relevant phage clones were obtained. Candidate phage clones were selected based on the following criteria: (i) highest frequency selection during the screening process, and (ii) highest reactivity in the ELISA test (e.g., Ph.D. 12mer 15: HFAYWWNGVRGP; Ph.D. 12 mer 18: AGHTTQFNSKTT; Ph.D. c7c 1: CNTGSPYEC; Ph.D. c7c 7: CSISSLTHC). One rabbit by each clone was subcutaneously immunized with 1 mL containing 2 × 10^12^ pfu in sterile PBS, mixed 1:1 with the adjuvant ISA 50 V (Seppic Montanide ™, France). Six immunizations were applied at intervals of 2 weeks each. The immunological reactogenicity and specificity of every antiserum from phage-immunized rabbits were evaluated by ELISA and Western blot, as already described before.

### Statistical analysis

Data were analyzed using the SAS/STAT 9.2 software (SAS Institute Inc., Cary, NC). Overall differences between groups were analyzed using ANOVA test. The mean value with respective standard deviation (SD) was calculated. A post-hoc analysis (Tukey’s test at *p* < 0.05) was performed after the overall analysis showed significant differences between all groups.

## Results

### Characterization of rabbit and bird anti-sporozoite sera by ELISA

**Figure 1:**
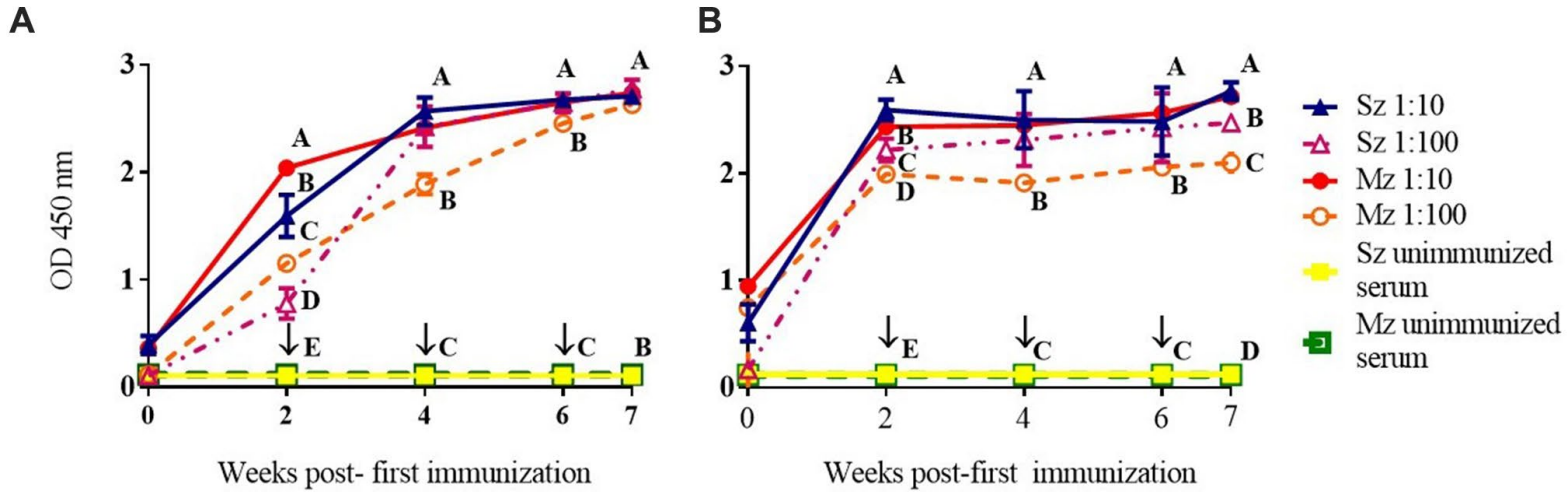
Immunization with whole-sporozoites of *E. tenella* to rabbits **(A)** and chickens **(B)**. The dynamics of antibody production in rabbits **(A)** and chickens **(B)** were assessed by ELISA against sporozoite (Sz) and second generation of merozoite (Mz) antigens, after immunization with sporozoites of *E. tenella*, at weeks 0, 2, 4, and 6 (↓). The rabbit and chicken antisera were diluted at 1:10 (▲) and 1:100 (△) againt Sz antigen, and the rabbit and chicken antisera were diluted at 1:10 (●) and 1:100 (○) against Mz antigen. Preimmune sera were diluted 1:10 and exposed to Sz (□) or Mz (■) antigens. Figures represent mean ODs (± SD). Different letters on the same data indicate statistical differences: *p < 0.05 (ANOVA followed by the Tukey post-hoc test).

Time-course of the reactogenicity of the anti-sporozoite sera from rabbits and birds immunized with whole-sporozoites of *E. tenella* was evaluated. Figure 1 shows the kinetics of *E. tenella* anti-sporozoite sera production in rabbits (Figure 1A) and birds (Figure 1B), assessed by its reactivity to sporozoite and merozoite; we can observe that rabbit and bird antisera cross-react to both asexual zoite stages in a similar pattern. Similar levels of antibody production were observed in rabbits and chickens; however, bird titers reached a plateau after 2 weeks, whereas rabbit titers did not reach a plateau until 4 weeks. Even though only sporozoites were used for immunization schedule, the immune response to the second generation of merozoites exhibited similar reactivity to the sporozoite antigen and followed the same dynamic pattern, indicating the presence of the same or cross-reacted antigens between both asexual zoite stages. Antisera diluted 1:100 showed better reactivity against the sporozoite than the merozoite (Figure 1B). At 7 week post-first immunization titers in rabbits against both asexual zoite stages are more homogenous than titers observed in birds at this same time indicating more affinity from rabbit antibodies toward both antigens.

### Reactivity in Western blot from two life-cycle stages of *Eimeria tenella* using anti-sporozoite sera

**Figure 2:**
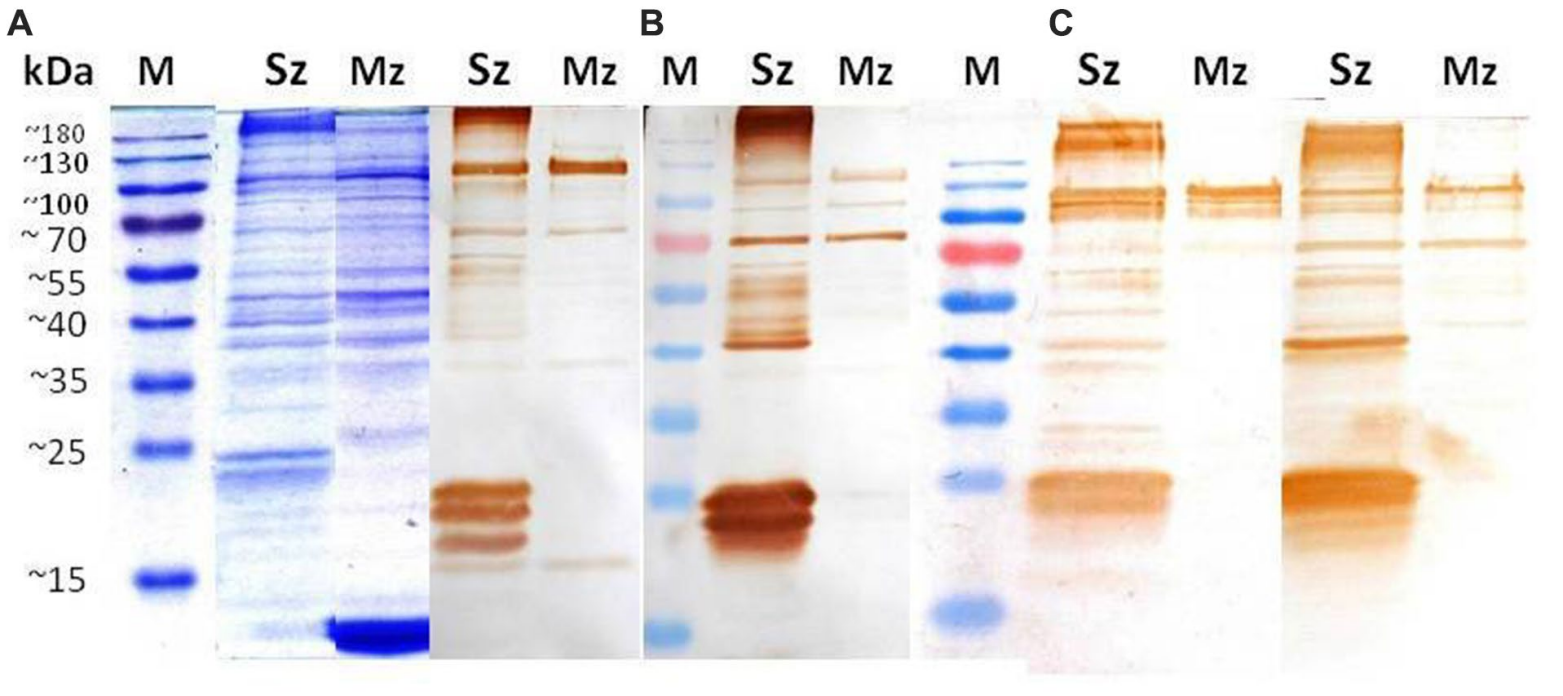
Identification of sporozoite and merozoite antigens by rabbit and chicken anti-sporozoite sera. Sporozoite (Sz) and merozoite (Mz) proteins were separated on a 12% SDS-PAGE and stained with Coomassie brilliant blue **(A)**. Western blot analysis with anti-sporozoite sera from two rabbits **(B)** and two birds **(C)**. The antisera were diluted 1/8000 in **(B)** and 1/2000 in **(C)**.

Reactogenicity of the anti-sporozoite sera from rabbits and birds immunized with whole-sporozoites of *E. tenella* against polypeptides of two asexual zoite stages of *E. tenella* was evaluated. Figure 2A shows the sporozoite and merozoite proteins separated on a 12% SDS-PAGE and stained with Coomassie brilliant blue. Although a complex protein profile of sporozoite and merozoite proteins was detected, it is possible to identify some common bands above 35 kDa. In the sporozoite, two specific distinct proteins below 25 kDa are evident, while in the merozoite, a band closer to 10 kDa is notoriously abundant. High molecular weight proteins seem shared among both asexual stages zoites but with some differences in concentration. The Western blot analysis with anti-sporozoite sera from rabbits (Figure 2B) and birds (Figure 2C) showed that both antisera recognized a similar polypeptide pattern in the sporozoite antigen, mainly polypeptides with molecular weight higher than 35 kDa, among which a 40 kDa protein is relevant. In the sporozoite, the most intense bands detected weighted 21 kDa, 23 kDa, and 24 kDa. In contrast, in the 2nd generation merozoite antigen, the antisera from rabbits or chickens indistinctly detected only a few high molecular weight antigens (approximately 70 kDa and 124 kDa). The most relevant here is that each rabbit antiserum had to be proportionally diluted four times more than the bird antiserums in order to obtain the clearest image of the PVDF membrane, which indicates a superlative reactogenicity of these antibodies toward both antigens subject to evaluation.

### Phage-display library screening with rabbit purified IgGs

**Figure 3:**
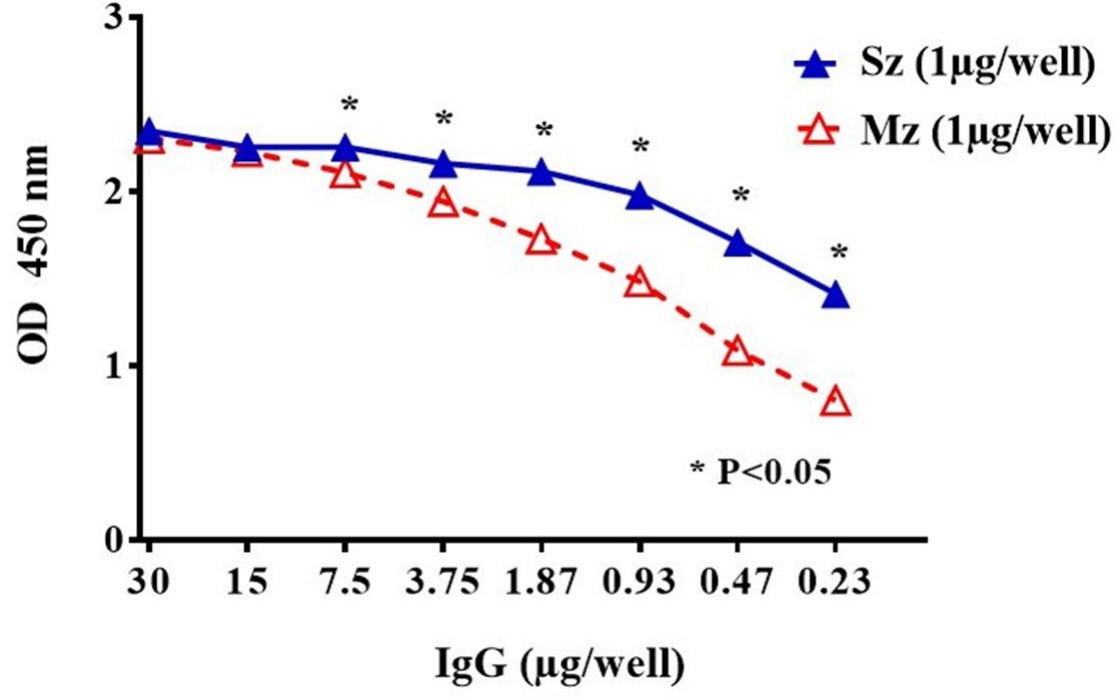
Indirect ELISA titration of purified rabbit anti-sporozoite IgGs to sporozoite (Sz ▲) and second generation of merozoite (Mz △). A two-fold IgG dilution was evaluated starting from 30 μg/well. Means in ODs (± SD) from duplicate wells are shown. Asterisks indicate statistical differences: *p < 0.05 (ANOVA, followed by Tukey post-hoc test).

In order to select recombinant Phage-display clones with specific affinity for rabbit anti-sporozoite antibodies, the IgG fraction was purified from the rabbit’s antisera by affinity chromatography with protein G sepharose. We diluted the purified IgG fraction from 30 μg/well to 0.23 μg/well to test by indirect ELISA their reactivity against the sporozoite or the 2nd generation of the Mz antigens. Figure 3 shows greater reactivity to the Sz antigen compared to the Mz antigen.

Using this same purified IgG fraction three screening rounds were performed for the 12-mer and cyclic 7-mer phage-display libraries. Table 1 shows the number of phage clones selected during each screening round. The selected phages of the 12-mer Ph.D. library increased from 2.0 × 10^5^ pfu in the first round to 6.9 × 10^6^ pfu in the third round. Similarly, phages obtained from the cyclic 7-mer Ph.D. library increased from 1.5 × 10^5^ pfu in the first round to 2.2 × 10^7^ pfu in the third round, indicating a selection of specific phage clones through the three screening rounds.

**Table 1:** Enrichment of phage clone output in three screening rounds.

	Ph.D. 12 mer (pfu/mL)			Ph.D. c7c (pfu/mL)		
Coating antibody	Eluted phage titer	Increase in phage titer (fold)	Amplified phage titer	Eluted phage titer	Increase in phage titer (fold)	Amplified phage titer
1st Pannig (10 μg/well)	2.0 × 10^5^	1.0	3.0 × 10^13^	1.5 × 10^5^	1.0	4.8×10^13^
2nd Pannig (5 μg/well)	2.8 × 10^6^	14.0	3.0 × 10^12^	3.3 × 10^5^	2.2	5.0 × 10^12^
3rd Panning (2.5 μg/well)	6.9 × 10^6^	34.5	N.A.	2.2 × 10^7^	146.7	N.A.

pfu plaque forming units; N.A. not amplified.

### Characterization of the phage-display selected clones

After the third screening round of each Phage-display random peptide libraries, 20 single phage clones were randomly selected and amplified. DNA sequencing was used to examine all of the selected clones. Tables 2, 3 provide the deduced amino acid sequences of the selected clones. DNA sequencing of the 12-mer Ph. D. library-selected phage clones revealed two immunodominant clones with the AGHTTQFNSKTT (7 times) and HFAYWWNGVRGP (8 times) peptide sequences. One clone with the amino acid sequence PNSAFWAGSER was obtained twice (Table 2). The clones selected from the cyclic 7-mer library were more diverse than those selected from the 12-mer library, four clones were selected twice, and one clone was selected three times (Table 3). All selected clones matched with one or two main proteins of *E. tenella* previously quoted in the NCBI gen bank. Some of these proteins are crucial in adhesion and invasion events of the sporozoite to the enterocyte, however, most of them have been described as hypothetical proteins.

**Table 2:** Peptide deduced aminoacid sequences on phage clones selected from 12 mer after three screening rounds.

12-mer Phage selected clones	*AA sequence	Identified protein, sequence ID, residues length and number of matches	Position/protein coverage
1	LHRGNEAVYAWP	Hypothetical protein, conserved [*E. tenella*] Sequence ID: XP_013228393.1 Length: 1103 Matches: 1	Position 553/6/7 (86%)
2,7,8,12,14,18,20	AGHTTQFNSKTT	Cullin homog, putative [*E. tenella*] Sequence ID: XP_013232647.1 Length: 208 Matches: 1	Position 54/6/7 (86%)
3	NRPDSAQFWLHH	Hypothetical protein, conserved [*E. tenella*] Sequence ID: XP_013228556.1 Length: 9860 Matches: 3	Position 1,326/7/9 (78%) Position 812/6/14 (43%)
11,17	GPNSAFWAGSER	Hypothetical protein, conserved [*E. tenella*] Sequence ID: XP_013233745.1 Length: 1116 Matches: 3	Position 76/6/7 (86%) Position 984/5/8 (63%)
6	FPVNNMQLWQVT	Hydroxymethyldihydropterin pyrophosphokinase-dihydropteroate synthase, putative [*E. tenella*] Sequence ID: XP_013228556.1 Length: 9860 Matches: 3	Position 262/8/11 (73%)
4,5,9,10,13,15,16,19	HFAYWWNGVRGP	Dynein beta chain, flagellar outer arm, putative [*E. tenella*] Sequence ID: XP_013232219.1 Length: 959 Matches: 1	Position 523/5/5 (100%)

* Consensus amino acid residues are highlighted in bold letters. Position and protein coverage for AA sequence of every selected phage clone on NCBI standard protein BLAST protein►protein sequence of the *Eimeria tenella* taxid 5,802.

**Table 3:** Peptide deduced aminoacid sequences on phage clones selected cyclic 7 mer library after three screening rounds.

Cyclic 7-mer Phage selected clones	*AA sequence	Identified protein, sequence ID, residues length and number of matches	Position/protein coverage
1,5	CNTGSPYEC	Microneme protein 4 [*E. tenella*] Sequence ID: CAC34726.2 Length: 2340 Matches: 10	Position 297, 991/5/8 (63%) Position 769, 810, 938, 124/6/9 (67%)
6	CMSTGLSSC	Thrombospondin type 1 domain-containing protein, putative [*E. tenella*] Sequence ID: XP_013235772.1 Length: 3774. Matches: 5	Position 3,575/7/9 (78%)
7,15,20	CSISSLTHC	Hypothetical protein, conserved [*E. tenella*] Sequence ID: XP_013233649.1 Length: 961. Matches: 3	Position 412/7/8 (88%)
8	CRSANIYTC	Hypothetical protein, conserved [*E. tenella*] Sequence ID: XP_013232311.1 Length: 1348. Matches: 1	Position 1,270/5/7 (71%)
9,16	CHPVSGQKC	Hypothetical protein ETH_00011430	Position 208;/6/7(86%)
10,18	**CLK**FWKPNC	Hypothetical protein, conserved [*E. tenella*] Sequence ID: XP_013228358.1 Length: 323 Matches: 1	Position 185/5/7(71%)
7′	**CLKL**GEKWC	Hypothetical protein ETH_00039830 [*E. tenella*] Sequence ID: XP_013231892.1 Length: 271 Matches: 1	Position 128/6/7 (86%)
10′	CA**KL**CLNNC	Asparaginyl-tRNA synthetase, putative [*E. tenella*] Sequence ID: XP_013227989.1 Length: 516 Matches: 1	Position 319/6/9 (67%)
11	CHQTKTKFC	Hypothetical protein ETH_00037435 [*E. tenella*] Sequence ID: XP_013229950.1 Length: 251 Matches: 1	Position 137/6/6 (100%)
13	CHNETQKMC	Dynein heavy chain 3, axonemal, related [*E. tenella*] Sequence ID: XP_013232337.1 Length: 1111 Matches: 1	Position 462/6/9 (67%)
17	CVGISALLC	Adenosine transporter, putative [*E. tenella*] Sequence ID: XP_013231508.1 Length: 443 Matches: 1	Position 197/7/9 (78%)
1′,3′	CPTNQHHLC	Regulator of chromosome condensation RCC1 (Precursor), related [*E. tenella*] Sequence ID: XP_013236104.1 Length: 812 Matches: 1	Position 26/6/8 (75%)
2′	CMNNFNITC	Hypothetical protein, conserved [*E. tenella*] Sequence ID: XP_013233414.1 Length: 1063 Matches: 1	Position 1/6/8 (75%)

* Consensus amino acid residues are highlighted in bold letters. Position and protein coverage for AA sequence of every selected phage clone on NCBI standard protein BLAST protein►protein sequence of the *Eimeria tenella* taxid 5,802.

### Genome sequence analysis of the phage-display selected clones

**Figure 4:**
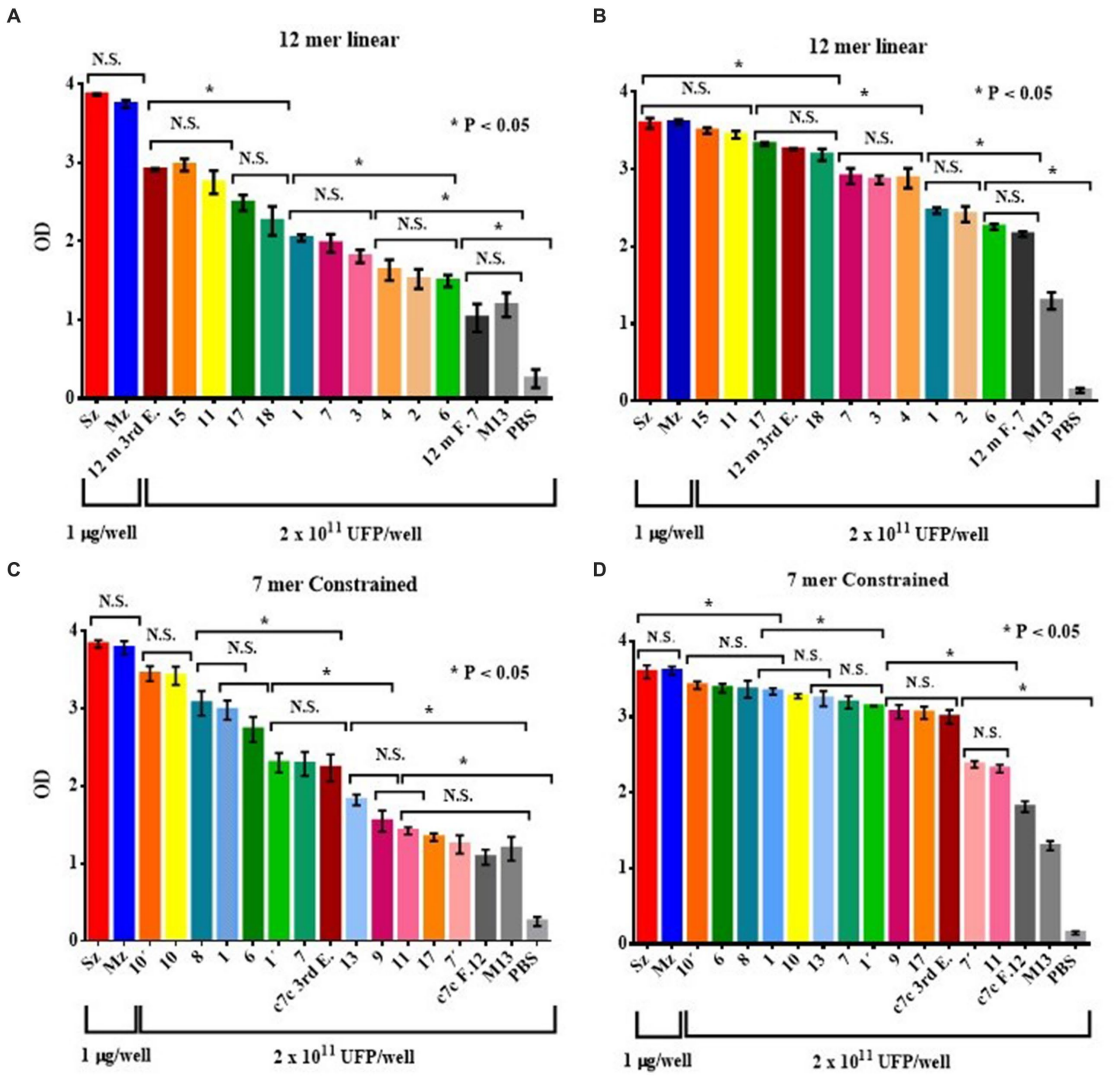
ELISA of the reactogenicity of rabbit **(A,C)** and chicken **(B,D)** anti-sporozoite sera to selected phage clones from the 12-mer **(A,B)** and c7c mer **(C,D)** libraries. The selected phage clones were tested by ELISA with rabbit and chicken anti-sporozoite sera. The wild-type phage M13mp19, an irrelevant phage clone for each library (selected with rabbit antisera anti-Pseudorabies virus, 12 m F.7 and c7c F.12), and PBS were used as negative controls. Reactivity of rabbit **(A)** and chicken antisera **(B)** against phage clones selected from the PhD 12-mer; rabbit antisera against selected clones from the c7c library **(C)**; and reactivity of the chicken antisera to selected clones from the c7c library **(D)** are shown. Bars represent mean ODs (± SD). NS, not significant. Asterisks indicate statistical differences: *p < 0.05 by ANOVA, applying the Tukey post-hoc test.

The *E. tenella* genome was BLAST screened with the sequence of every selected peptides. The peptides obtained from the 12 mer library found clear identities (Table 2). The AGHTTQFNSKTT peptide matched with a Cullin homolog. It was found that the HFAYWWNGVRGP peptide has an identity with the Dynein beta chain. The phage clone sequence GPNSAFWAGSER showed three matches with the Elongation Factor 2 (EF-2). The peptides obtained from the cyclic 7-mer library also found matches in the *E. tenella* genome (Table 3). The CNTGSPYEC clone had ten matches with the extracellular domains of EtMIC4’s epidermal growth factor-like repeats. Furthermore, the amino acid sequence of the phage clone CMSTGLSSC matched the thrombospondin type-I repeats of EtMIC4. In contrast, the CSISSLTHC clone showed several matches with a hypothetical protein conserved in *E. tenella*. Finally, as seen in Table 3, at least three 7-mer peptides share the CLK and KL motifs; interestingly, two of these phage clones showed the highest reactivity in the ELISA test (Figure 4C).

### Reactivity of the phage-display selected clones to anti-sporozoite rabbit and bird sera

The anti-sporozoite sera from rabbits and Leghorn chicks were used to test the recognizing level of every Phage clone selected from each Phage display random peptide libraries. Figure 4 shows the antigen reactivity of the selected Phage clones to rabbit and bird anti-sporozoite sera evaluated by indirect ELISA. Most of the selected phage clones interacted specifically with anti-sporozoite sera and had greater OD values than negative control sera (Figure 4). Every Phage clone showed different level of reactogenicity toward anti-sporozoite sera. Although with some differences almost all Phage clones showed reactogenicity in the same order to both anti-sporozoite sera, it indicates that the sporozoite has immunodominant antigens recognized by both species.

### Phage-display selected clones induce anti-sporozoite and anti-merozoite antibodies

**Figure 5:**
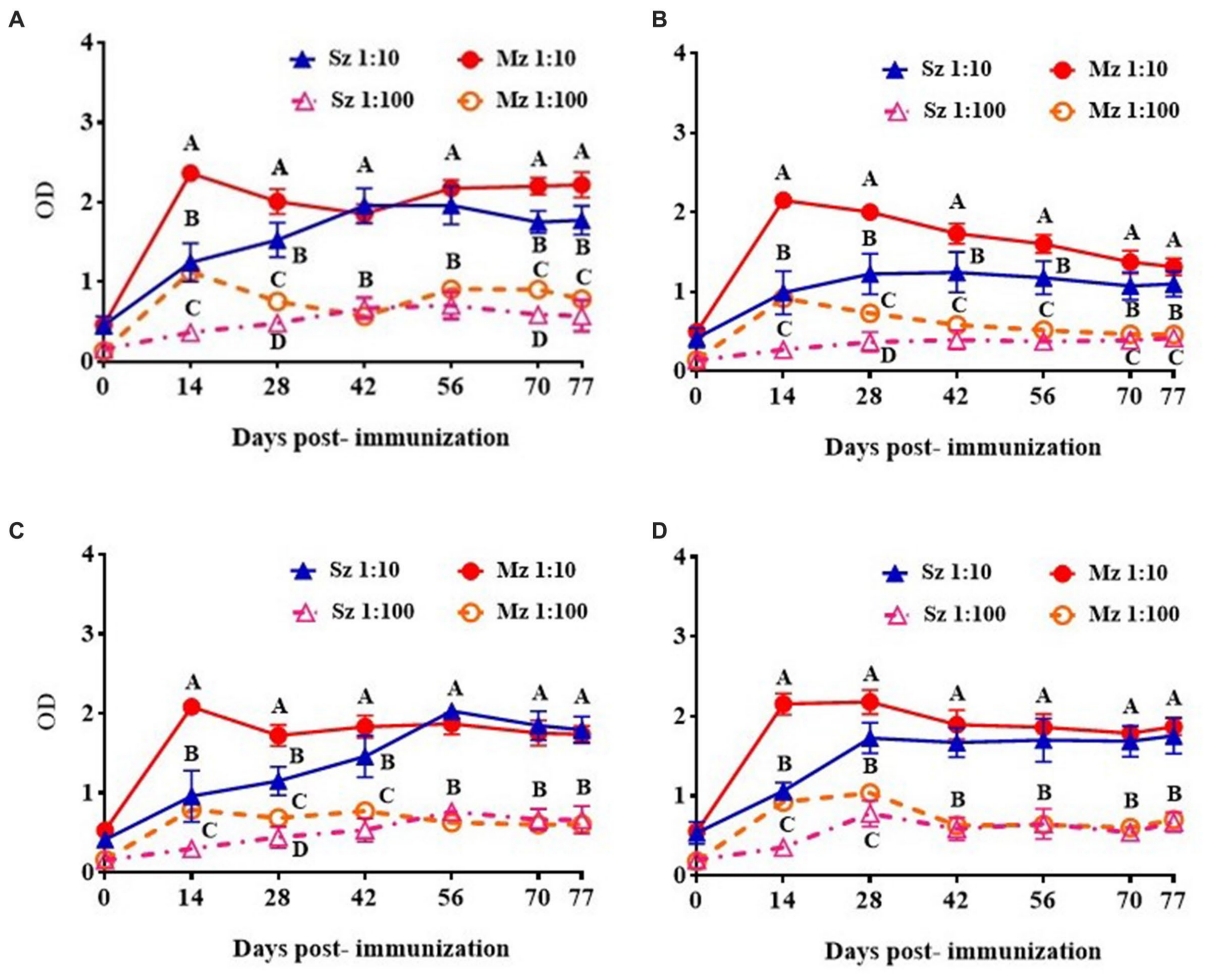
ELISA reactivity of rabbit antiserum of selected clones to sporozoite and second generation of merozoite antigens. Rabbit anti-phage clones serum was diluted 1/10 and 1/100 and tested against sporozoite and merozoite antigens. The antiserum response from different phage clones is shown: **(A)** Phage clone 1, C-NTGSPYE-C; **(B)** Phage clone 7, C-SISSLTH-C; **(C)** Phage clone 15, HFAYWWNGVRGP and **(D)** Phage clone 18, AGHTTQFNSKTT. Figures represent mean ODs values with respective ± SD. Different letters on the same date indicate statistical differences: *p < 0.05 (ANOVA followed by Tukey post-hoc test).

If the peptide clones selected with the anti-sporozoite antibodies represent mimotopes present in *E. tenella* antigens, these phage clones will be able to induce antibodies against *E. tenella* antigens. In that sense, four Phage clones were selected and used to immunize one rabbit each. Every anti-clone rabbit serum was used to assess the recognition of the sporozoite and merozoite native antigens. These four Phage clones were selected for rabbit immunization based on their (i) ELISA reactivity to anti-sporozoite antibodies, (ii) frequency of clone selection, and (iii) bioinformatic similarity with *E. tenella* proteins. In this way, phage clones 1 and 7 of the Ph.D. c7c library, and phage clones 15 and 18 of the Ph.D. 12 mer library were selected for rabbit immunization (Table 2). Figure 5 shows the time course reactogenicity of the antiserum from every rabbit immunized with the selected phage clones to both asexual zoite stages of *E. tenella*. Although with different time course reactivity against every life cycle of *E. tenella* antigen, overall the rabbit anti-clone serums showed high titer recognizing both antigens tested, it was observed a time course light tendency to recognice more to Mz than Sz antigen, however at last of the immunization shedule the reactogenicity of every anti-clone serum agains both antigens was indistinghishable.

### Recognition of *Eimeria tenella* sporozoite and 2nd generation of merozoite antigens by anti-phage serums

**Figure 6:**
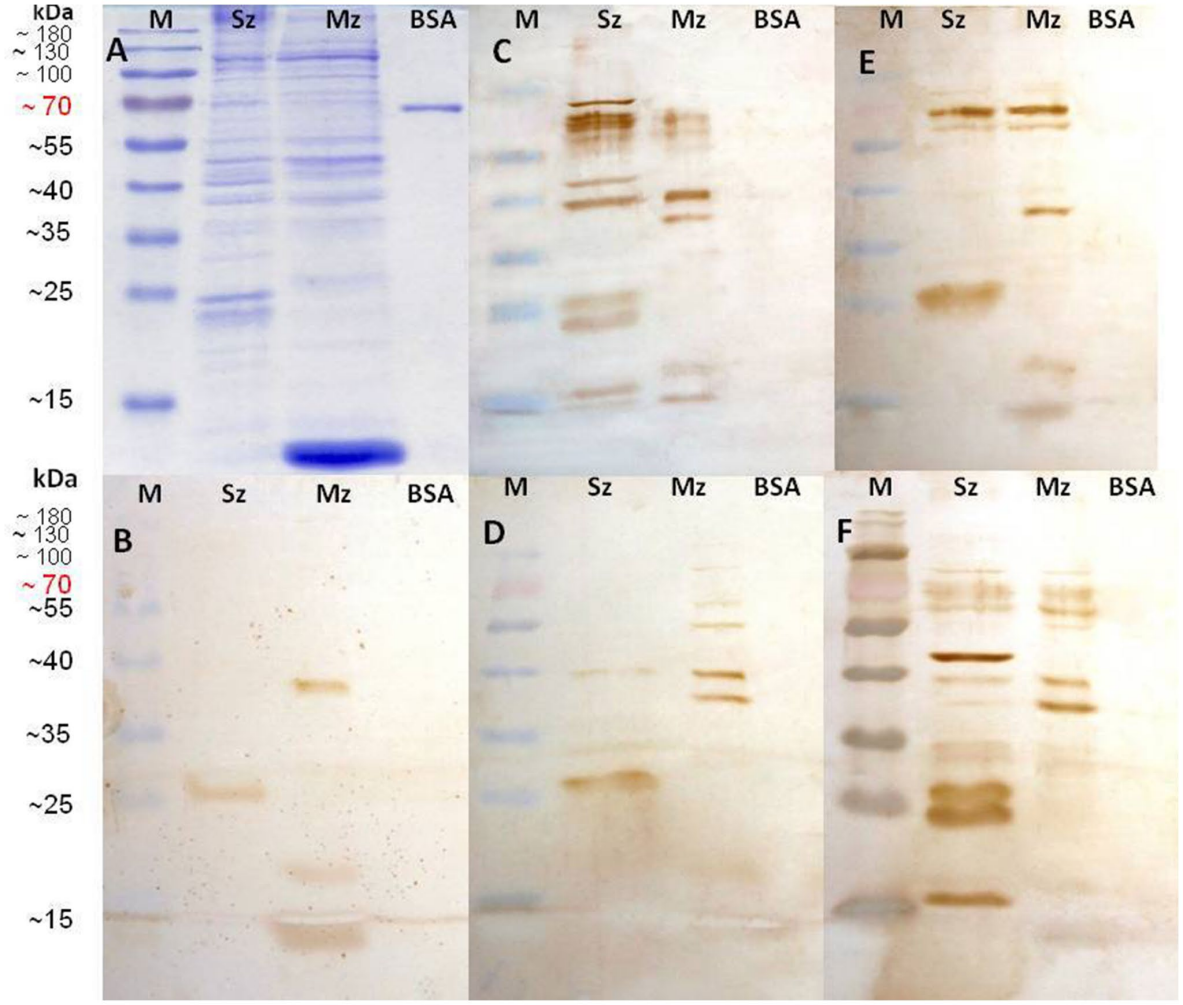
Identification of sporozoite and second generation of merozoite antigens by every rabbit anti-phage clone serum. Sporozoite (Sz), Merozoite (Mz), and Bovine Serum Albumin (BSA) proteins were separated on a 12% SDS-PAGE and stained with Coomassie brilliant blue **(A)**. Western blots are shown with wild-type M13 phage rabbit antisera used as negative control **(B)**; rabbit anti-phage clone 1 (C-NTGSPYE-C) serum **(C)**; rabbit anti-phage clone 7 (C-SISSLTH-C) serum **(D)**; rabbit anti-phage clone 15 (HFAYWWNGVRGP) serum **(E)**, and rabbit anti-phage clone 18 (AGHTTQFNSKTT) serum **(F)**.

The reactivity level and specific polypeptide recognition of every rabbit anti-phage serum to Sz and Mz antigens was investigated by Western blot. Figure 6 shows the reactivity of the anti-phage 1 (Figure 6C), anti-phage 7 (Figure 6D), anti-phage 15 (Figure 6E), and anti-phage 18 (Figure 6F) serums. Figure 6A shows both *E. tenella* asexual zoite stages protein profile and BSA resolved by 12% SDS-PAGE electrophoresis. It was a control group for every PVDF membrane studied. Figure 6B shows the reactivity of the antiserum pool from two rabbits immunized with wild-type M13 used as a negative control.

All anti-phage serums notably recognized both Sz and Mz antigens (Figure 6). The anti-M13 negative control sera recognized bands of approximately 38 kDa, 20 kDa, and 13 kDa in the Mz proteins and only a ≈ 25–27 kDa range band in the Sz extract. These bands were also detected in all blots tested with all anti-phage serums (Figure 6). The anti-phage 1 serum detected prominent bands of 78 kDa, 68 kDa, 48 kDa, and 40 kDa in the Sz proteins (Figure 6C, lane Sz). In addition, a pair of approximately 23 and ≈25–27 kDa antigens (as was already seen with M13 antiserum) were recognized. Two bands of 68 kDa and 60 kDa were recognized in the Mz proteins; both share the same molecular weight with antigens detected in the sporozoites. A band of 40 kDa was observed, which is also shared in the Sz antigens (Figure 6A).

The phage clone 7 antiserum (Figure 6D) detected at the Mz extract a pattern of bands closely similar to the anti-phage 1 serum. In contrast, very few bands were detected in the Sz proteins. A light band of 40 kDa (also present in the Mz) and a band of ≈25–27 kDa (present in the anti-M13 sera). The 40 kDa antigen was detected in the Sz and Mz proteins by the anti-phage 1 and anti-phage 7 serums. Anti-phage 15 (Figure 6E) and anti-phage 18 (Figure 6F) serums detected a similar antigenic pattern in the Mz proteins and a pair of bands of approximately 68 kDa and 70 kDa. These bands were also detected in the Sz antigen. A specific band of 40 kDa was detected in the Sz and Mz proteins with the anti-phage 18. This antiserum recognized a prominent band of 45 kDa. This same antiserum also recognized bands of 34, 33, 27, 24, and 17 kDa in the Sz extract (Figure 6F, lane Sz). Although with different rank of reactivity and molecular mass, the four anti-phage clone serums recognized more bands in the Sz and Mz antigens than recognized by the sera from rabbits immunized with a wild-type M13 strain that was used as a negative control.

## Discussion

Several studies have shown that chickens become clinically resistant to artificial trickle infections with *E. tenella* oocysts (37–39). When antisera of these chickens have been used to probe immunoblots on sporozoites and merozoites of *E. tenella*, a similar pattern of antigens has been identified on both asexual zoite stages (35, 36, 38). We discovered that rabbits and hens inoculated subcutaneously with a vaccine of complete *E. tenella* sporozoites induce a humoral response that detects the same particular antigens in both asexual life-cycle phases of *E. tenella*. Our Western blot observation is consistent with previous studies, showing that antibodies generated against one asexual zoite stage cross-react with antigens from another asexual zoite stage (10, 26).

Although rabbits and chickens responded equally to both *Eimeria* antigens, rabbit antisera showed a much greater antibody response to both antigens than antisera derived from SPF White Leghorn birds. Antiserum from a rabbit had to be diluted 1:8,000, whereas antiserum from a Leghorn chicken only had to be diluted 1/2,000. This has been done to improve the signal-to-noise ratio of the images of Western blot membranes. This is consistent with previous observations that sporozoites injected subcutaneously are more immunogenic than oocysts inoculated naturally (orally) to infected chickens (40). Furthermore, it has been shown that heterologous hosts, such in this case the rabbits, recognize more critical epitopes of the sporozoites than those recognized by natural hosts (40–42). In addition, it should be noted that the evolutionary distance between birds and rabbits permits the production of unique, specific antibodies against, for example, antigens of common pathogens in birds.

Pathogen-specific monoclonal or polyclonal antibodies can be employed in phage display combinatorial library screenings to find recombinant specific peptides of immunodominant epitopes with high affinity to these antibodies. Due to the scarcity of information on the nature of *E. tenella* protective antigens, we used rabbit anti-sporozoite antibodies as a probe in our study to discover key immunogenic epitopes that could be protective against *E. tenella* infection (6, 8, 11). Indirect ELISA testing revealed that the majority of the 20 selected clones from each library strongly reacted to anti-sporozoite rabbit sera, showing that the selected recombinant peptide binds to a particular antibody against the *E. tenella* sporozoite. The DNA sequencing of the selected phage clones revealed that some phage clones were identical and had been selected many times. In the panning of random Phage-display libraries, the relative abundance of monospecific antibodies in a polyclonal mixture of an immune serum is expected to direct phage clone selection (17). The frequency of the selected recombinant peptides here suggests that these phagotopes are immunogenic and diverse. The peptides selected from the cyclic 7-mer library (suitable for selecting conformational epitope) were more varied than those obtained from the lineal 12-mer library.

Typically, many *Eimeria* antigens identified as vaccine candidates often play a role in host/parasitic interaction, most likely because these antigens are naturally exposed during parasite invasion/replication mechanisms and are thus accessible targets for the host immune response (7, 16, 33, 43–46). Proteins released from micronemes, organelles situated at the apical tip of apicomplexan parasites, whose contents are crucial for parasite gliding, motility, and adhesion to host cells, as well as entrance into infected cells, are among the most intensively studied (6, 16, 44, 47–49). In order to identify and determine the function of the target *Eimeria* antigens, Sasai et al. (50) employed monoclonal antibodies (mab’s) against recombinant EtMIC2 protein. These mabs first detected the micronemes of the sporozoite and merozoite of *Eimeria* sp. In another study, Witcombe et al. (51) demonstrated that the persistence of maternal protection in offspring chicks was connected with the reactivity of protective maternal (polyclonal) IgY antibodies with a high-weight molecular TRAP (thrombospondin-related anonymous protein)-family microneme protein termed EmTFP250.

The epidermal growth factor-like modules (EGF-like) and thrombospondin type 1 domain (TSP-1) motifs are found in high repetition in the EmTFP250, a feature also observed in other Apicomplexa parasites such as Plasmodium and Toxoplasma (49). EmTFP250 is related to *E. tenella* microneme protein 4 (EtMIC4) (52), and it is believed that, as a protein complex together with EtMIC5, it plays a crucial function in host cell adhesion and invasion (53). Moreover, EtMIC4, unlike the other *E. tenella* microneme proteins studied to date, appears to be present both on the sporozoite surface and within the micronemes in a constitutive manner (47, 54, 55). EtMIC4 contains adhesive domains conserved in higher eukaryotes, which include the EGF-like and the TSP-1 motifs (52, 54, 55). Interestingly, the genomic screening of *E. tenella* NCBI-Blast with the Ph.D. c7c mer selected peptide sequences found multiple matches with the EGF-like and TSP-1 modules of EtMIC4. The results obtained in the present study suggest that the screening strategy of the Ph.D. c7c mer library increased the possibility of identifying conformational peptides with cysteine-restricted motifs, such as EGF-like and TSP-1 of EtMIC4 (54, 55). These findings demonstrated that these peptides were immunodominants when the *E. tenella* intact sporozoites were inoculated as an immunogen in an animal different from a natural host (40, 55).

Comparative analysis of the phagotope CSISSLTHC disclosed matches with a conserved hypothetical protein of *E. tenella*. Blake et al. (15) previously reported that at least 70% of *E. tenella* genes are currently classified as uncertain functions or are well characterized as conserved hypothetical proteins. Until recently, only a few genes of the conserved hypothetical proteins in *Eimeria* sp. have been examined and tested for function and immunogenicity (15). A single-family of two 7mer peptides share the consensus motif C-LKLxxxN-C; both were selected in the same phage-display random panning (Table 3), and the phage clone CAKLCLNNC showed the highest reactivity in the indirect ELISA, likely indicate that they are mimics of an immunodominant epitope of the *E. tenella* sporozoite.

The use of phage displays as a means of mapping epitopes in Apicomplexa could be an innovative strategy. However, the intricacy in the protein composition of every stage zoite of *E. tenella* makes mapping of epitopes represented by the mimotopes somewhat complicated (9, 44, 47). Further studies using random Phage display libraries involving heterologous antibodies against different life-cycle stages of *E. tenella* as ligands are required.

Liu et al. (33) analyzed the proteome of second-generation merozoites using antisera from chickens previously infected with *E. tenella*. However, these antibodies only detected a small number of microneme and merozoite surface antigens in addition to other housekeeping proteins like enolasa, beta-tubulin, and heat shock protein 70. Recent research by Liu et al. (45) used antisera from *E. tenella*, *E. acervulina*, and *E. maxima* to perform a Western blot of two-dimensional gels containing sporozoite proteins from every *Eimeria* species. Fifty-four immunodominant proteins were identified in *E. tenella*, and 18 ortholog proteins were identified among the three *Eimeria* species. Five of the 18 ortholog proteins shared sequence similarity of more than 93% and were identified as common immunodominant antigens; these proteins included elongation factor 2 (EF-2), ubiquitin-conjugating enzyme domain-containing protein (UCE), 14–3-3 protein, glyceraldehyde-3-phosphate dehydrogenase (GAPDH) and transhydrogenase.

Some of the *E. tenella* native protein sequences available in GenBank are similar to the deduced oligopeptide sequences of the phage clones selected from the Ph.D. 12 mer library; however, only two of them have been previously identified as immunodominant epitopes of the *E. tenella* sporozoites (6, 45). For example, the phagotope AGHTTQFNSKTT matched with cullin homolog putative protein of *E. tenella*, a protein that recruits particular targets to ubiquitin ligase in a multisubunit protein complex beneficial for ubiquitination (45, 56). This phagotope may be connected to UCE, a protein previously discovered as a common immunodominant antigen for *Eimeria* sp. by Liu et al. (45). Otherwise, the most reactive phagotope in the ELISA assay (Figure 4A) matched with dynein beta chain, flagellar outer arm, a putative protein of *E. tenella*, which creates force toward the minus ends of the microtubules in the inner membrane (44), the latter protein is essential for *Eimeria* invasion of enterocytes, while the former protein has already been identified as an immunodominant peptide by Liu et al. (44).

The phagotope GPNSAFWAGSER, which ranked second in indirect ELISA reactivity, matched with a putative protein that contain ankyrin-like repeats of *E. tenella*, in some Apicomplexa members this protein contributes to regulate the conoid stability, motility and cell invasion (57). There is no prior evidence that these proteins are involved in coccidiosis protection (6, 7). The phage clone GPNSAFWAGSER was also related to the putative *E. tenella* elongation factor-2 (EtEF-2) protein, which, according to Liu et al. (45), is a common immunodominant antigen in *E. tenella*, *E. acervulina*, and *E. maxima*.

While this particular phage clone had not previously been characterized and tested as a vaccine candidate, Panebra and Lillehoj (58) recently tested a recombinant vaccine platform with the same type of protein (EaET-1) in a priming/challenge trial, with promising results for cross-protective immunity against *Eimeria* sp. Lately, Liu et al. (46) described the elongation factor 1-β, putative, partial (EF-1 β), another protein from this group, as an immunodominant antigen common to sporozoites of *E. acervulina*, *E. tenella*, and *E. necatrix*. These findings support the vaccination potential of different elongation factor antigens for coccidiosis and open up new avenues for developing multivalent vaccines against *Eimeria* sp., which is crucial for the poultry industry.

In the search for immunodominant antigens, the selected peptide clones are expected to represent epitope mimics or mimotopes present in *E. tenella* antigens (reviewed in Adda et al. [17]). One way to corroborate this is to obtain antibodies against these peptides and then evaluate their immune reactogenicity to native *E. tenella* antigens. Our ELISA and Western blot data demonstrated that each rabbit anti-phage serum successfully detected specific antigens in the *E. tenella* sporozoite and 2nd generation of merozoite antigens. The latter supports the idea that the selected peptides correspond to true mimotopes, evidencing the immunogenic and protective potential of these phagotopes.

Interestingly, these mimotopes are presumably present in more than one life-cycle stage of *E. tenella*, indicating that either every mimotope is expressed by both parasite stages or the antiserum recognizes more than one mimotope paralogue. Antiserums from rabbits immunized with phagotope CNTGSPYEC or AGHTTQFNSKTT recognized more antigens in the sporozoite than in merozoite. All of these recognized antigens concatenate well with antigens initially identified by rabbit antibodies used to screen both Ph.D. libraries (Figure 2B).

In the present study, the rabbit antiserums against both phagotopes recognized more than one antigen band, indicating that some proteins in the crude preparation of sporozoite and merozoite of *E. tenella* are under proteolytic activity like it would be doing *in vivo* infection (47). Typically, micronemal and rhoptry proteins are often proteolytically cleaved during biogenesis and post-exocytosis to allow sporozoite cell identification and attachment to host cell membranes (14, 44, 47). Fragments generated from each *E. tenella* denaturized protein (Sz and Mz) could be keeping the same epitopes in scattered small pieces of different molecular weights. Each of these fragments could be maintaining these epitopes similarly as they were in the original quaternary structure of *the E. tenella* native protein, which would explain this multivalent recognition. Another possible explanation is that the anti-clone serum cross-reacts with the same specific antigen located in both life-cycle phases of *E. tenella*, and this antigenic determinant is in several polypeptides with different molecular mass. Further identification of biological targets in the intact *E. tenella* sporozoites and 2^nd^ generation of merozoites with every anti-clone serum might be illuminating.

Our observations confirm that mimotopes can produce native antigen-specific antibodies in active vaccination programs against distinct pathogen targets (19, 21, 22, 29). Curiously, different anti-phage serums reacted to the same prominent band in the sporozoite, which had a molecular weight of approximately ≈25–27 kDa (Figure 6). This antigenic band was also found in our immunostained membranes of Western blot probed with rabbit and chicken anti-sporozoite sera; however, this polypeptide was not observed in blottings of Sz and Mz antigens tested with antisera of chickens naturally (orally) immunized with *E. tenella* by Constantinoiu et al. (36), despite this band (≈25–27) was evident in their CBB stained gels. In the early 1990s, surface antigens like TA4, a 25-kDa polypeptide encompassing 17 and 8 kDa, were identified (6, 7). TA4 was later identified as the sporozoite-specific glycosyl-phosphatidylinositol (GPI) anchored surface antigen (SAG)1, which can bind cultured epithelial cells and may play a role in parasite attachment to the enterocyte prior to invasion (59). When used as a recombinant protein immunogen, DNA vaccine, or a *Salmonella typhimurium*-vectored vaccine, SAG1 has been shown to induce partial protective immunity (6, 14, 60). Previously, using two-dimensional electrophoresis and mass spectrometry, de Venevelles et al. (43) identified a sporozoite antigen TA4 precursor with a predicted molecular weight of 25.04 kDa but an experimental molecular weight of 26.86 kDa, which is more similar to the molecular mass of the band that we identified here. The latter helps us better understand the changes reported by several researchers throughout time regarding the specific molecular weight of this highly immunogenic peptide observed in our blots. In the meantime, further studies are being conducted to evaluate the immunogenicity of all these characterized phagotopes in priming/challenge trials.

## Conclusion

Both phage display libraries used here successfully discovered novel candidates of B- cells antigens from *E. tenella*. This attractive reverse immunology approach leads to identifying high immunogenic *E. tenella* sporozoite mimotopes and their coding DNA sequence. The isolated clones revealed relatedness to dynein beta chain, flagellar outer arm putative, cullin homolog putative, elongation factor 2, microneme 4 protein of *E. tenella*, and a conserved hypothetical protein of *E. tenella*. The phagotopes were recognized by antisera from rabbits immunized with complete sporozoites of *E. tenella*, indicating their potential use as immunotopes.

## Data availability statement

The original contributions presented in the study are included in the article/supplementary material, further inquiries can be directed to the corresponding author.

## Ethics statement

The animal study was reviewed and approved by The FMVZ-UNAM’s Institutional Review Board for Husbandry and Care in Animals (CICUA) examined and authorized the animal study through the Ph.D. internal board.

## Author contributions

MJ-E and RA-M: conceptualization, methodology, software, and writing original draft preparation. MJ-E, GT-I, DG, and AG-V: visualization and investigation. MJ-E, LL, and GT-I: data curation and statistical analysis. MJ-E, GT-I, and RA-M: funding acquisition, reviewing, and editing. GT-I and RA-M: supervision. All authors contributed to the article and approved the submitted version.

## Funding

The research was partly supported by funds provided by USDA-NIFA Sustainable Agriculture Systems, Grant No. 2019–69012- 29905. Title of Project: Empowering United States Broiler Production for Transformation and Sustainability USDA-NIFA (Sustainable Agriculture Systems): No. 2019–69012-29905. This work was supported by the National Autonomous University of Mexico (UNAM)–Technological Innovation, Research Projects Support Program (PAPIIT). Project Number: IN217022 “Alternative Strategies for Prophylaxis of Avian Coccidiosis in México”.

## Conflict of interest

The authors declare that the research was conducted in the absence of any commercial or financial relationships that could be construed as a potential conflict of interest.

## Publisher’s note

All claims expressed in this article are solely those of the authors and do not necessarily represent those of their affiliated organizations, or those of the publisher, the editors and the reviewers. Any product that may be evaluated in this article, or claim that may be made by its manufacturer, is not guaranteed or endorsed by the publisher.

## Footnotes

^1^ https://blast.ncbi.nlm.nih.gov/Blast.cgi
